# Dynamics of Skin Mycobiome in Infants

**DOI:** 10.3389/fmicb.2020.01790

**Published:** 2020-07-28

**Authors:** Ting Zhu, Yuan-Yuan Duan, Fan-Qi Kong, Carlos Galzote, Zhe-Xue Quan

**Affiliations:** ^1^Ministry of Education Key Laboratory for Biodiversity Science and Ecological Engineering, Institute of Biodiversity Science, School of Life Sciences, Fudan University, Shanghai, China; ^2^AP Skin Testing Center, Johnson & Johnson China Ltd., Shanghai, China; ^3^Johnson & Johnson International (Singapore) Pte. Ltd., Manila, Philippines

**Keywords:** infant, skin mycobiome, individual variation, *Malassezia*, internal transcribed spacer

## Abstract

Understanding the microbial community structure of the human skin is important for treating cutaneous diseases; however, little is known regarding skin fungal communities (mycobiomes). The aim of the present study was to investigate the features of and variations in skin fungal communities during infancy in 110 subjects less than 6 months of age. Skin samples were obtained from the back, antecubital fossa, and volar forearm, while physiological parameters including transepidermal water loss, pH, surface moisture, and deep layer hydration were evaluated. Skin fungal diversity decreased after the first three months of life. Differences in fungal community composition were greater among individual infants than among the three skin sites in the same individual. Inter- and intra-individual variation were similar and lower, respectively, than the variability between two samples obtained 12 weeks apart, from the same site in the same subject, suggesting low stability of fungal communities on infant skin. Skin physiological parameters showed little correlation with skin fungal community structure. Additionally, *Malassezia* was the most represented genus (36.43%) and *M. globosa* was the most abundant species in *Malassezia* with its abundance decreasing from 54.06% at 0–2 months to 34.54% at 5–6 months. These findings provide a basis for investigating the causative fungi-skin interactions associated with skin diseases.

## Introduction

The skin along with its resident microbes, provide the first line of defense, protecting the body from external environmental attack (Grice et al., 2009). Many studies including The Human Microbiome Project, an ongoing effort to sequence the so-called second genome of humans, aim to characterize skin microbes (Grice and Segre, 2012). The skin fungal community (mycobiome), therefore, plays an important physiological role in protecting the skin from infection by external pathogenic microorganism and powerful modifiers of host biology; however, studies examining these roles are insufficient compared with those on the skin bacterial counterpart (Naik et al., 2012; Belkaid and Tamoutounour, 2016; Jo et al., 2016; Lynch and Hsiao, 2019). Studies on infant skin commensal fungus are even rarer (Findley et al., 2013; Jo et al., 2016). This represents a key knowledge gap, because the status of early skin microbes could have a profound impact on skin functionality and vulnerability in future disturbances (Ward et al., 2017; Lynch and Hsiao, 2019; Zhu et al., 2019).

The skin bacterial community of healthy individuals has been well studied using modern sequencing-based approaches, particularly for adults (Grice et al., 2009; Ying et al., 2015; Zhu et al., 2019); in this population, the bacterial community composition is significantly influenced by intrinsic and extrinsic host factors, such as age, skin site, make-up, and washing habits (Grice and Segre, 2011; McDowell et al., 2016; Byrd et al., 2018; Chen et al., 2018; Ross et al., 2018; Bouslimani et al., 2019), and are stable for up to 2 years (Dorrestein et al., 2016). Bacteria, though constituting the majority, are not the only microorganisms contributing to the human skin microbial ecosystem (Oh et al., 2014; Byrd et al., 2018). Skin mycobiomes, representing less than 10% of skin microbes (Oh et al., 2014), are associated with numerous skin diseases, including psoriasis and atopic dermatitis (Fry and Baker, 2007; Zhang et al., 2011; Takemoto et al., 2015; de Hoog et al., 2017). Despite the importance of fungi in dermatology, most literature discussing the skin mycobiome pertains only to pathogenic fungi and not normal skin fungal communities. The composition of a skin mycobiome was reported to be more similar than that of bacterial communities across core adult body sites, regardless of physiology (Byrd et al., 2018). Alternatively, a study examining the mycobiomes of one-month-old infants, showed that they differed by body site, and highlighted the large differences between individuals (Ward et al., 2018). Even so, studies describing the early skin fungal community composition remain insufficient, and evidence on the extent of changes in infant skin over time is lacking.

Both culture- and DNA-sequence-based identification of human skin-associated fungi have revealed the importance of *Malassezia* (formerly known as *Pityrosporum*, predominating at core body and arm sites) in terms of its relative abundance and function (Saunders et al., 2012; Findley et al., 2013; Cabanes, 2014; Moallaei et al., 2018). Human skin harbors *Malassezia* from birth, especially at sebaceous sites (e.g., the forehead, back, and behind the ears) (Lee et al., 2006; Byrd et al., 2018). Although *Malassezia* accounts for a low relative abundance on infant skin (∼2% under first month), it has relatively large abundance on adult skin (75.0–90.7%) (Sugita et al., 2010; Ward et al., 2018). *Malassezia* is a member of the normal skin flora, yet is also related to human autoimmunity and skin diseases (Dawson, 2007). Quantitative analyses of human skin *Malassezia* composition indicated that *Malassezia globose* and *Malassezia restricta* have an abundance advantage over other *Malassezia* species and are affected by individual factors (Lee et al., 2006; Gao et al., 2010; Sugita et al., 2010). Nonetheless, information on the community status of *Malassezia* on healthy skin is currently insufficient.

In this study, we analyzed the fungal community composition including *Malassezia* subspecies, of 110 infants aged 0–6 months, in relation to specific skin physical parameters. We also randomly selected 39 infant subjects to undergo reassessment at 6-week intervals (baseline, week 6 and week 12) to observe the temporal changes associated with infant skin.

## Materials and Methods

### Ethics Statement

This study was conducted in compliance with all International Conference on Harmonization (ICH) Good Clinical Practice Guidelines, including ICH E6 (Dixon, 1998). The research (protocol, informed consent agreement, release of photographs, recruitment materials [advertisements, telephone script, etc.]), and all addenda were approved by the Institutional Review Board of Beijing Children’s Hospital. Written, informed consent was obtained from the parents prior to enrollment of the infants in the study. All sample information was anonymous. Throughout the experiments, we adhered to the safety standards of the institution.

### Subjects

Information for each subject is provided in Supplementary Table S1. Totally 130 infants aged 0–6 months old (A1, A2, A3, A4, and A5 represent 0–2, 2–3, 3–4, 4–5, and 5–6 months, respectively) were enrolled in and completed the sampling. Subjects were all healthy full-term infants born in the hospitals and were of Chinese descent. Parents/caregivers (≥18 years old) of the infants expressed their willingness to follow study instructions and provided signed informed consent. Further inclusion criteria were as follows: (i) parents/caregivers, and infants had no history of asthma or unusual/hypersensitive/allergic response to skin care products; (ii) infants neither had history of dermatologic diseases nor were on any type of antibiotic treatment. The subjects of this study were the same as those in Duan et al. (2019) and detailed information can be found in this previously published article (Duan et al., 2019). Subjects were instructed to wash with only water during the experimental period and not to wash the specific body sites for 2 h prior to sampling. A total of 130 subjects were included in the sampling, of which 110 subjects were ultimately retained for analysis, including 52 female infants and 58 male infants (Supplementary Table S1). Of the 110 subjects, 33 lived in suburban areas of Beijing, and the remainder lived in urban areas of Beijing. The division of suburban and urban areas is based on the administrative division of Beijing.

### Sampling

During June and July (summer, baseline) of 2013 in Beijing, China, all 130 infants were sampled, and 43 of them were randomly selected as a group, in which the infants were cleansed with water only, for sampling after 6 and 12 weeks (Group 3 in the previous publication) (Duan et al., 2019). Sampling was completed by November 2013. Environmental assessment (temperature and humidity of outdoor/indoor environments) in Beijing over the sampling timeframe can be found in the previous publication (Duan et al., 2019). Simultaneously, we measured the physiological parameters of the volar forearm skin, including surface moisture (Corneometer CM 825; Courage & Khazaka Electronics, Köln, Germany), pH (Skin-pH-Meter PH 900; Courage & Khazaka), transepidermal water loss (TEWL; Vapometer; Delfin Technologies, Kuopio, Finland), and deep layer hydration (Moisture Meter-D; Delfin Technologies); these data were previously published (Duan et al., 2019). Samples were obtained by swabbing a 2 cm × 2 cm region from three skin sites, including the back, antecubital fossa, and volar forearm using sterilized polyester swabs soaked in a solution of 0.15 M NaCl and 0.1% Tween 20 (Paulino et al., 2006; Fierer et al., 2008). Blank swabs were included for each sampling time point to ensure no contamination during sampling and sample processing.

### Sample Processing

DNA was extracted within 24 h of sample collection, using the MoBio PowerSoil DNA Isolation kit (Qiagen, Valencia, CA, United States) as described previously (Ying et al., 2015; Zhu et al., 2019). Nested PCR was used to amplify the fungal internal transcribed spacer 1 (ITS1) using dual-barcoded primers; NSA3 (5′-AAACTCTGTCGTGCTGGGGATA-3′) and NLC2 (5′-GAGCTGCATTCCCAAACAACTC-3′) were used for the first round of amplification (Martin and Rygiewicz, 2005; Toju et al., 2012). Each 25 μL reaction contained 12.5 μL Ex Taq Premix v.2.0 (Takara, Dalian, China), 1 μL of each forward and reverse primer (2.5 μM), 5 μL template DNA, 1 μL bovine serum albumin (20 mg/mL), and 4.5 μL ddH_2_O. PCR reaction conditions were: 94°C for 5 min; 30 cycles at 94°C for 30 s, 50°C for 45 s, 72°C for 60 s; and 72°C for 10 min. NSI1 (5′-NNNNNNNNNNNN GATTGAATGGCTTAGTGAGG-3′) and 58A2R (5′-NNNNNNNNNNNN CTGCGTTCTTCATCGAT-3′) with 12-nt barcodes were used as primers for the second round of PCR (Martin and Rygiewicz, 2005; Hamady et al., 2008), which was similar to the first round, save for 2.5 μL of product from the first round being used as a template in the second round, and the number of amplification cycles was 25. Negative controls for the PCR reaction were conducted simultaneously with each PCR amplification. PCR-amplified products were pooled and purified using the AxyPrep DNA Gel Extraction kit (Axygen, Tewksbury, MA, United States) according to the manufacturer’s instructions, and then quantified with the dsDNA HS Assay (Invitrogen, Darmstadt, Germany) and used for library construction for Illumina sequencing with the LTP Library Preparation kit (KAPA Biosystems, Boston, MA, United States). Sequencing data were obtained using an Illumina Miseq platform (San Diego, CA, United States) using the 2^∗^300 paired-end protocol.

### Raw Data Analysis

The quality of post-sequencing data was verified using FastQC^1^. Sequences were processed using the Quantitative Insights Into Microbial Ecology (QIIME) v.1.8 platform (Caporaso et al., 2010). The orientation of paired-end reads was first adjusted according to the primer sequence. To prevent the effects caused by variable lengths of PCR products from different fungi, reads were trimmed, with only the first 200 nt of reads retained, as all bases with a Phred score <20 in the last 50 nt. The complete dataset was chimera-checked against the UNITE database using USEARCH61. Sequences were assembled by placing the reverse complement of Read 2 in front of Read 1 and were assigned to samples according to the barcode. Next, subjects with samples of depth less than 2,400 reads (20/130 subjects and 4/43 subjects sampled three different times) were removed from further analysis. Similar sequences were assigned to operational taxonomic units (OTUs) based on a 95% identity threshold with USEARCH61 using QIIME script “*pick_open_reference_otus*.” Taxonomy was assigned to each representative sequence using the Ribosomal Database Project (RDP) Classifier (Wang et al., 2007). Microbial diversities within, and between communities, were evaluated by alpha (Chao1, Shannon and observed species) and beta (Bray-Curtis distances) diversities, respectively. A co-occurrence network was constructed with the top 20 genera. The correlation between each genus was confirmed by the R package “*psych*” with *R* ≥ 0.6 and *p* < 0.01. Data visualization was conducted using Gephi software (Bastian et al., 2009).

Sequences related to the order Malasseziales were separated for more sophisticated processing using the QIIME script “*pick_de_novo_otus*” function based on the 95% identity threshold. A custom species-level reference dataset was established, with 12 *Malassezia* ITS1 sequences for taxonomy assignment, relating to the most abundant *Malassezia* OTUs in the skin mycobiome. This dataset can cover more than 95% of the infant’s skin *Malassezia spp.* population. Samples were rarefied to 500 sequences per sample and subsampled for principal coordinates analysis (PCoA) according to age, site, and gender to characterize the *Malassezia* community.

### Statistical Analysis

Significantly higher alpha diversity values between different sample parameters were identified with the two-tailed *t*-test. Analysis of variance (ANOVA) was used to assess OTU abundance regarding different groups of attributes, to identify associated taxa. Analysis of similarities (ANOSIM) was used to identify differences between various sample attributes. A correlation test between skin parameters and fungal taxa was performed with the Pearson’s correlation test. The relationship between fungal community composition and skin physical parameters was evaluated with the Mantel test. The Wilcoxon test was used to compare the average Bray-Curtis matrix of different groups. Except for the Wilcoxon test, which was performed using R software, all statistical analyses were performed in QIIME. *P*-values for multiple comparisons with ANOVA and Pearson’s correlations were corrected using the Bonferroni method.

### Data Availability

Sequence data generated in the present study and the custom *Malassezia* references were deposited in the National Omics Data Encyclopedia (NODE^2^) under accession number OEP000722 (Project ID).

## Results

### Fungal Communities on Infant Skin

**FIGURE 1:**
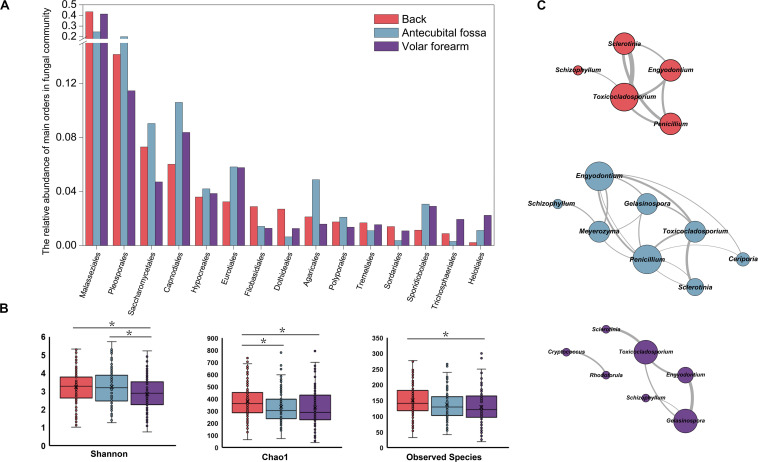
Fungal profile on three sites of human infant skin. **(A)** Fifteen fungal orders with relative abundance >1% on three skin sites. **(B)** Alpha diversity of skin mycobiomes on three skin sites; **(C)** The co-occurrence networks of top 20 fungal genera (presented in Supplementary Table S2) on three skin sites (*R* ≥ 0.6 and *p* < 0.01). Red stands for the back, blue for antecubital fossa, and purple for volar forearm; *: *p* < 0.05.

The samples with ≥2400 sequences depth from 110 subjects (Supplementary Table S1) showed that the phyla Ascomycota (49.95%) and Basidiomycota (48.83%) were similarly represented on the back, antecubital fossa, and volar forearm skin. A total of 61 orders were represented in the samples, with 28 from Ascomycota and 31 from Basidiomycota; fifteen of these orders had an abundance over 1.00% at all three sites (Figure 1A). Within the genera in Ascomycota, *Alternaria* (10.73%), *Cladosporium* (7.00%), *Candida* (5.91%), and *Aspergillus* (2.46%) were the most abundant (>2.00%), whereas in Basidiomycota, *Malassezia* (36.43%), and *Cryptococcus* (3.21%) were the most highly represented (Supplementary Table S2).

The main factor contributing to variations in fungal community composition was skin site (*R* = 0.046, *p* = 0.001), followed by subject age (*R* = 0.030, *p* = 0.011), and gender (*R* = 0.029, *p* = 0.001). Delivery type did not have statistically significant influence on the mycobiome of infants’ skin within the first six months. Among the three skin sites, mycobiomes of the back showed the highest alpha diversity (Figure 1B and Table 1). Fungal orders, including Malasseziales and Agaricales, showed differences in abundance among the three sites, with the former most prevalent on the back and volar forearm and the latter most prevalent on the antecubital fossa (Figure 1A, *p* < 0.05). At the genus level, *Malassezia* (Malasseziales) and *Schizophyllum* (Agaricales) were similarly represented (*p* < 10^–5^). The mutual promotion between fungal genera was more pronounced on the antecubital fossa, where *Malassezia* showed the lowest relative abundance (average 24.48%) compared to the back (average 43.43%) or volar forearm (average 41.37%). Notably, *Malassezia* and *Alternaria*, the two main groups on the skin, did not participate in such co-occurrence interactions (Figure 1C and Supplementary Table S2).

**TABLE 1:** ANOVA comparisons of alpha diversity index according to attributes of 110 subjects at baseline.

**Attribute**	**Shannon**	**Chao1**	**Observed species**
Site	Antecubital fossa > Volar forearm (*p* < 0.05); Back > Volar forearm (*p* < 0.05)	Back > Antecubital fossa (*p* < 0.05); Back > Volar forearm (*p* < 0.05)	Back > Volar forearm (*p* < 0.05)
Age	NS	A1 > A3, A4 (*p* < 0.05); A2 > A3, A4, A5 (*p* < 0.05)	A1 > A3, A5 (*p* < 0.05); A2 > A3, A4, A5 (*p* < 0.05)
Gender	NS	NS	NS
Residence	suburban > urban (*p* < 10^–5^)	suburban > urban (*p* < 10^–5^)	suburban > urban (*p* < 10^–5^)
Delivery type	NS	NS	NS

*A1, 0–2 months; A2, 2–3 months; A3, 3–4 months; A4, 4–5 months; A5, 5–6 months; NS, non-significant.*

Alpha diversities of A1 (representing age 0–2 months) and A2 (representing age 2–3 months) were significantly higher than those for the older groups (A3 [3–4 months], A4 [4–5 months], and A5 [5–6 months]) in the fungal community (Table 1). There was no difference in the distribution at the order level across age groups, however, the abundance of the genus *Alternaria* differed between A1 and A4 (16.80 and 5.85%, respectively; *p* < 0.05). Males and females showed differences in the abundance of orders Malasseziales (41.81 and 30.42%, respectively; *p* < 0.05) and Sordariales (0.33 and 1.65%, respectively; *p* < 0.05), but no difference was observed in alpha diversity. The relative abundance of order Pleosporales was higher in infants born by vaginal delivery than in infants born by cesarean section (18.30 and 11.83%, respectively; *p* < 0.05), particularly for the genus *Alternaria* (13.79 and 7.43%, respectively; *p* < 0.05). Genera *Candida* (8.43 and 4.83%, respectively; *p* < 0.05) and *Aspergillus* (3.88 and 1.85%, respectively; *p* < 0.05) were more abundant in infants living in suburban communities compared to those in urban settings, with the former having significantly higher alpha diversity (Table 1).

The Mantel test was conducted to investigate the relationship between fungal community composition and skin physical parameters; only the A5 from all age groups, sites, genders, and delivery types, showed a significant relationship with skin pH. Meanwhile, skin deep layer hydration, transepidermal water loss, and skin moisture were unrelated to fungal community structure (Supplementary Table S3).

### Changes of Infant Skin Fungal Community Composition Within 12 Weeks

**FIGURE 2:**
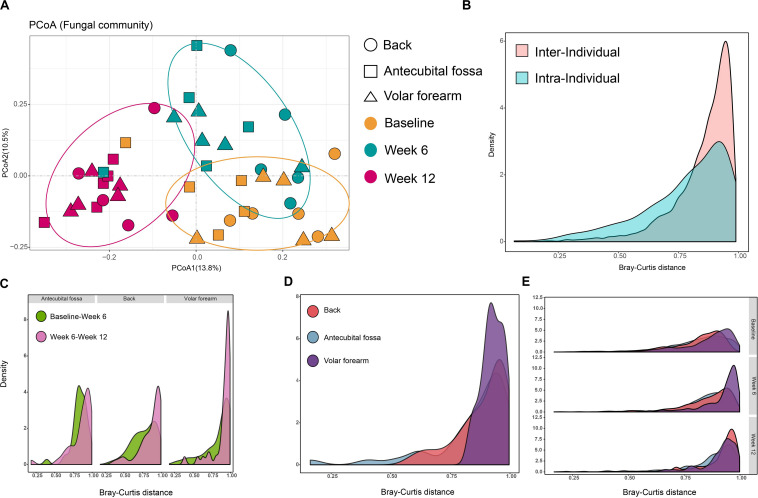
Temporal stability of infant skin mycobiomes. **(A)** PCoA analysis of samples that were taken at baseline, week 6, and week 12 from 39 subjects. Samples were subsampled according to age, site, sampling time, and gender. **(B)** Density of inter-individual Bray-Curtis distances (from same site in different subjects) and intra-individual Bray-Curtis distances (from different sites in the same subject); **(C,D)** Density of Bray-Curtis distance among samples from infants 6 weeks **(C)** and 12 weeks **(D)** apart in age and from the same site in the same subject; **(E)** Density of inter-individual Bray-Curtis distance of the same site among different subjects during three phases. Data for the three phases and three sites are presented.

We examined 39 subjects (Supplementary Table S1) at three sampling timepoints (baseline, week 6, and week 12) to assess the dynamics of infant skin mycobiomes. As expected, sampling time strongly influenced the composition of skin mycobiomes (*R* = 0.146, *p* = 0.001) and was a factor clearly affecting clustering in PCoA (Figure 2A and Supplementary Figure S1).

Intra-individual Bray-Curtis distances between different sites on the same subject at the same sampling time (baseline, week 6 or week 12), and inter-individual Bray-Curtis distances between the same sites on different subjects at the same sampling time, were calculated. Inter-individual distances were found to be greater than intra-individual distances (*p* < 10^–5^; Figure 2B), indicating that the differences in fungal communities between individual infants were greater than the differences between different sites from one individual. Furthermore, the distances between two samples collected 12 weeks apart in age (baseline to week 12) from the same site on the same subject, were greater than intra- (*p* < 0.05), but not inter-individual distances. This further illustrates that the differences in infant skin fungal communities, induced by a 12-week interval, may be as large as the difference between individuals. Individual specificity was applicable in the skin fungal community of infant populations, however, skin site specificity may be less obvious on infant skin. The changes in different sampling times between skin sites showed that there was less variation in the first six weeks, from baseline to week 6, compared to week 6 to week 12, particularly on the back and volar forearm (Figure 2C). The volar forearm also had the highest variation from baseline to week 12 (Figure 2D). Inter-individual distances differed significantly between the three sampling times (*p* < 0.05), increasing from baseline to week 12 on the back and antecubital fossa, but not on the volar forearm (Figure 2E).

Comparisons of alpha diversity indices revealed a significant difference in Chao1 among the three sampling times (304 for week 12, 352 for week 6, and 368 for baseline; *p* < 0.05). The top 20 abundant genera were evaluated by ANOVA to determine their temporal stability at the three times (Table 2 and Supplementary Table S4) and *Malassezia* demonstrated variable distribution at the three timepoints, with a decrease in abundance from baseline to week 12. *Cryptococcus* and *Filobasidium* also showed clear differences between any two successive timepoints, with the highest abundance at week 6, however, no difference was observed between baseline and week 12. Changes in the relative abundance of *Aspergillus*, *Meyerozyma*, *Stemphylium*, *Sterigmatomyces*, *Alternaria*, and *Toxicocladosporium* occurred between week 6 and week 12, with the highest abundance at week 12 and no significant differences between baseline and week 6. In contrast, *Rhodotorula* and *Gelasinospora* showed the greatest changes between baseline and week 6, with no significant change between week 6 and week 12. *Fusarium* was stable with an increase in abundance from baseline to week 12. No significant correlation was observed between fungal community structure and skin physical parameters at the three sampling timepoints.

**TABLE 2:** Difference in relative abundance of 20 fungal genera between phases.

**Genera**	***P*-value of ANOVA**			**Relative abundance**		
	**Baseline vs Week 6**	**Baseline vs Week 12**	**Week 6 vs Week 12**	**Baseline**	**Week 6**	**Week 12**
*Malassezia*	0.001	0.000	0.001	0.361	0.243	0.126
*Alternaria*	NS	NS	0.027	0.117	0.078	0.146
*Cladosporium*	NS	NS	NS	0.080	0.122	0.086
*Candida*	NS	NS	NS	0.049	0.051	0.040
*Aspergillus*	NS	0.000	0.000	0.023	0.007	0.089
*Cryptococcus*	0.000	NS	0.005	0.014	0.072	0.027
*Rhodotorula*	0.049	NS	NS	0.013	0.045	0.027
*Fusarium*	NS	0.031	NS	0.011	0.025	0.036
*Meyerozyma*	NS	0.002	0.001	0.010	0.006	0.047
*Erysiphe*	NS	NS	NS	0.003	0.023	0.013
*Stemphylium*	NS	0.004	0.010	0.001	0.004	0.031
*Schizophyllum*	NS	NS	NS	0.021	0.011	0.000
*Filobasidium*	0.022	NS	0.034	0.001	0.028	0.002
*Sterigmatomyces*	NS	0.005	0.006	0.000	0.001	0.028
*Gelasinospora*	0.045	NS	NS	0.019	0.000	0.007
*Penicillium*	NS	NS	NS	0.015	0.007	0.005
*Pleurotus*	NS	NS	NS	0.000	0.005	0.017
*Toxicocladosporium*	NS	0.009	NS	0.001	0.004	0.014
*Pyricularia*	NS	NS	NS	0.012	0.003	0.000
*Debaryomyces*	NS	NS	NS	0.002	0.008	0.004

*NS, non-significant.*

#### Malassezia

**FIGURE 3:**
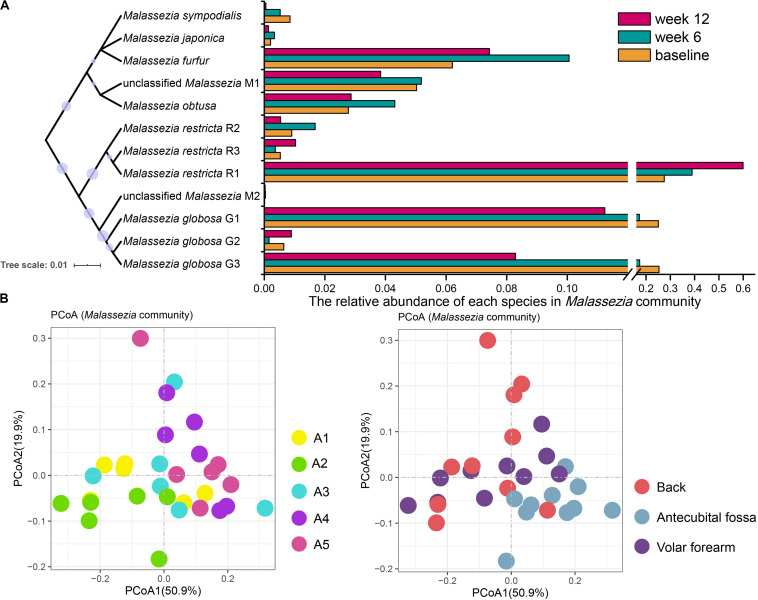
*Malassezia* community of infant skin mycobiomes. **(A)** The relative abundance of species in *Malassezia* communities at baseline, week 6 and week 12. A Neighbor-Joining phylogenetic tree of *Malassezia* references used in this study. **(B)** PCoA analysis of *Malassezia* community of 110 subjects at baseline. Samples were subsampled according to age, site, sampling time, and gender. A1, 0–2 months; A2, 2–3 months; A3, 3–4 months; A4, 4–5 months; A5, 5–6 months.

We also conducted species-level analyses of *Malassezia* on samples (6/110) with *Malassezia* sequences less than 500 excluded from the subspecies analysis. Results show that *M. globosa* (48.17%), *M. restricta* (29.45%), *M. furfur* (6.85%), *M. obtusa* (3.6%), *M. sympodialis* (1.23%), and *M. japonica* (0.3%) were detected, with *M. globosa* and *M. restricta* together accounting for 70%. We then performed a phylogenetic analysis of subspecies (G1, G2, and G3 for *M. globosa* and R1, R2, and R3 for *M. restricta*; Figure 3A) and identified unclassified *Malassezia* type (M1, 5.77%).

Among the 110 subjects at baseline, *M. restricta* R1, *M. globosa* G1, and *M. globosa* G3 were the most prevalent subspecies. *M. restricta* R1 was highly represented on the antecubital fossa (antecubital fossa: 38.82%, volar forearm: 24.21%, and the back: 20.68%; *p* < 0.05), whereas *M. globosa* G3 was less abundant (antecubital fossa: 16.40%, volar forearm: 26.03%, and the back: 26.84%; *p* < 0.05). Moreover, the *M. globosa* G1 population fluctuated with age, with the abundance in A2 significantly different from the other age groups (A2: 37.75%, A1: 27.24%, A3: 22.55%, A4: 17.82%, A5: 16.80%; *p* < 0.05). Age and site (*R* = 0.057 and 0.052, respectively; *p* = 0.001, Figure 3B) were the strongest factors influencing changes in the *Malassezia* community composition at baseline. A1, A2, and A3 samples clustered together and were distinct from A4 and A5 in PCoA (Figure 3B). *M. globosa* showed a decreasing trend with age (A1 and A2: 54.06%, A3: 44.47%, A4: 37.72%, and A5: 34.54%), meanwhile gender had no statistical correlation with the variation of the *Malassezia* composition at baseline.

Samples taken at baseline, week 6, and week 12 from 39 subjects were analyzed for temporal stability in *Malassezia* subspecies abundance (Figure 3A). *M. globosa* G3 retained more advantage at baseline than week 12 (baseline: 25.37%, week 12: 8.29%; *p* < 10^–6^), particularly on the volar forearm (baseline: 28.57%, week 12: 4.69%; *p* < 10^–3^). The relative abundance of *M. restricta* R1 changed at each sample time (baseline: 27.51%, week 6: 38.93%, and week 12: 59.52%; *p* < 0.05), and was higher at week 12 than at baseline or week 6 on the volar forearm (baseline: 21.08%, week 6: 29.08%, and week 12: 63.75%; *p* < 0.01).

## Discussion

Infant skin mycobiomes are important constituents of young humans’ microbial ecosystem (Costello et al., 2012; Fierer et al., 2012; Byrd et al., 2018). While there have been studies on the prevalence of pathogenic species, less is known about the general status of early human skin fungi (Findley et al., 2013; Kong et al., 2017; Ward et al., 2017). This study investigated the skin mycobiome in healthy infants through the differences among skin mycobiomes and simultaneously, the degree of change after a short time. The major fungal genera on infant skin (i.e., *Malassezia*, *Cladosporium*, and *Candida*) were detected in all analyzed samples and were also described as important fungi of residential surfaces (Amend et al., 2010; Adams et al., 2013). Moreover, specific fungal species, that are not commonly found on the skin of healthy children and adults were detected in this study. For instance, *Schizophyllum*, which was reported to exist in the human oral environment and indoor surfaces (Schmidt, 2007; Peters et al., 2017), was abundant on the antecubital fossa of infants (average 2.58%). Moreover, *Alternaria*, which is a common fungus that freely floats in the air, and is present on adult skin at a relatively low abundance (Takahashi, 1997; Zhang et al., 2011; Findley et al., 2013), was prevalent on infant skin (average 10.73%). Compared with other age groups, the micro-habitat on infant skin differs significantly with higher moisture content, pH, and skin conductance, as well as lower lipid content and microbial biomass (Cotterill et al., 1972; Duan et al., 2019). These factors may provide a certain colonization space for air-borne or contact-borne microbes on infant skin that are not commonly observed on the skin of older individuals.

We found that skin sites and ages of subjects were the major factors accounting for the diversity and composition of skin mycobiomes and this was consistent with what has been previously reported for adults (Gao et al., 2010; Findley et al., 2013; McDowell et al., 2016; Sugita et al., 2016). However, the low *R*-value (<0.1) determined here, and in a previous report (Ward et al., 2018), indicates that the skin site has less impact in babies than in adults (Grice et al., 2009; Findley et al., 2013; McDowell et al., 2016). In adults, the skin mycobiome is dependent on body site rather than on individuality (Findley et al., 2013), meanwhile in infants, we found that differences between the same sites of different individuals were significantly greater than differences between different skin sites of the same subjects, as previously reported for newborn babies (Ward et al., 2018). Adult skin sites can be categorized by their physiological characteristics as oily, moist or dry, with the highest fungal diversity observed in oily skin (Byrd et al., 2018). Among the three skin sites in the present study, the mycobiome from the back showed the highest alpha diversity. This result indicates that the oily environment that has proven advantageous to fungal species is already developed on the backs of infant within only months of birth. Regarding age, a study has reported a trend toward increasing Shannon index values over time during the first month of life on forehead skin (Ward et al., 2018). We observed that this trend persisted for the first three months of life (A1 and A2) before the diversity declined, particularly on the back. These results suggest that the diversity of skin fungi does not steadily increase on an early human body, as environmental and individual diversifications have a greater impact than age.

Additionally, infants living in suburban areas, with significantly higher relative abundance of *Candida* and *Aspergillus*, showed higher mycobiome alpha diversity than did those living in urban areas (generally considered to have a higher level of hygiene). Although this is contrary to a previous report (McCall et al., 2020), there are some studies that support the notion that human hygiene may reduce skin bacterial diversity (Council et al., 2016; Ross et al., 2018; McCall et al., 2020). The environment accessible to infants (aged 0–6 months in this study) is significantly different from that of humans of other age categories, and this may cause differences in the effect of urbanization on mycobiome. Alternatively, the effects of birth mode (cesarean section or vaginal birth) on skin mycobiomes was not statistically relevant in the present study, however, was relevant in a study of infants younger than one month (Ward et al., 2018). Further, the effect of birth mode was observed on the skin bacterial community of 10-year-old children (Zhu et al., 2019), highlighting that bacterial communities are more environmentally sensitive, and more tightly integrated with the host’s experience and phenotype, than are mycobiomes (Lynch and Hsiao, 2019; Tong et al., 2019).

A relatively high content of *Malassezia* in males compared to females was also observed in this study, which corresponded to results reported for adults (Leung et al., 2016), however, are different from that in older children (Jo et al., 2016). The rate of sebum excretion is significantly lower in boys than in girls and is higher in males than in females after the age of 15 (Cotterill et al., 1972). This pattern is consistent with the pattern observed in the relative contents of *Malassezia* (Jo et al., 2016; Leung et al., 2016). Therefore, the lipid level of males may be higher than that of females even for infants, and this may cause the relatively high content of *Malassezia* in males. In addition, no correlation was observed between skin mycobiomes and physicochemical indicators by statistical analysis in the present study.

When assessing the stability of skin mycobiomes over 6 and 12 weeks, we found that the change in skin mycobiomes after 12 weeks was similar to the inter-individual variation with no significant difference, indicating that infant skin mycobiomes are less stable than those of adults, and are more susceptible to seasons and aging (Findley et al., 2013; Tong et al., 2019). The outdoor temperature and relative humidity during the three sampling times were 5–28°C and 10–50%, respectively (Duan et al., 2019), during summer, autumn, and early winter. As the *R*-value was small when examining the influence of age, changes in seasonal or external environmental conditions were considered to be the major influential factors causing differences among the three sampling timepoints. The primary taxa susceptible to external environmental conditions were *Malassezia*, *Aspergillus*, *Meyerozyma*, *Stemphylium*, *Filobasidium*, and *Sterigmatomyce*, which exhibited significant changes at the three sampling times. Moreover, certain fungi did not change significantly across the three sampling timepoints and exhibited short-term stability, including *Cladosporium*, *Candida*, *Erysiphe*, *Schizophyllum*, *Penicillium*, *Pleurotus*, *Pyricularia*, and *Debaryomyces*.

*Malassezia* is a group of common lipophilic fungi on the skin that require skin lipids for growth and are implicated in common dermatologic conditions such as seborrheic dermatitis (Fry and Baker, 2007; Cabanes, 2014; Moallaei et al., 2018). *Malassezia* was predominant in all body sites examined in the present study (36.4%, primarily composed of *M. restricta* and *M. globosa*) and its relative abundance continued to reduce from baseline to week 12 with the decrease of environmental temperature (Duan et al., 2019). Alternatively, *Malassezia* reportedly remained stable across the four seasons on adult skin (Tong et al., 2019). In the current study, the average *Malassezia* relative abundance of 36.4% was higher than that in newborn babies (2.0%) (Ward et al., 2018), similar to that in children aged 7 to 14 years (36.3%) (Jo et al., 2016), and lower than that in adults (75.0–90.7%) (Gupta and Kohli, 2004; Akaza et al., 2010; Nagata et al., 2012; Leung et al., 2016). This shows that *Malassezia* can rapidly accumulate, in relative abundance, in the months after birth. *M. globosa* and *M. restricta* are the main *Malassezia* species on human skin (Jo et al., 2016; Leung et al., 2016), including that of infants enrolled in the present study, and their relative abundance changes with age (Cabanes, 2014). Further subspecies analysis in our study, indicates that the main type of *M. restricta* was *M. restricta* R1. Other studies illustrated that *M. globosa* is also the most abundant *Malassezia* species among children under 14 years old in the United States and in Japanese females between 10 and 18 years old (Sugita et al., 2010; Jo et al., 2016). The abundance of *M. globosa* in this study exhibited a decreasing trend (from 54.06 to 35.54% in *Malassezia*) with age and sampling times, particularly *M. globosa* G1 and G3. The relative abundance of *M. restricta* R1 increased with sampling times, however, not with age. *M. restricta* and *M. globosa* have different lipase activity and preference of the composition of lipid source (Ro and Dawson, 2005; Wu et al., 2015). The secretion state of skin lipid in different seasons and ages may be the reason for the change in the composition of *Malassezia*.

In summary, this study revealed the features and dynamics of the skin fungal community during infancy. Inter-individual differences were higher than differences among three skin sites in the same individual. Skin fungal diversity decreased after the first three months of life. *Malassezia* was the most highly represented genus with 36.4%, while *M. globosa* was the most prevalent species, however, its abundance decreased with age. These findings provide novel insight into skin fungal communities of infants and serve as a basis for investigating the fungal etiologic agents of cutaneous diseases.

## Data Availability Statement

The datasets presented in this study can be found in online repositories. The names of the repository/repositories and accession number(s) can be found at: http://www.biosino.org/node, OEP000722.

## Ethics Statement

The studies involving human participants were reviewed and approved by the Institutional Review Board of Beijing Children’s Hospital. Written informed consent to participate in this study was provided by the participants’ legal guardian/next of kin.

## Author Contributions

Z-XQ, Y-YD, F-QK, and CG conceived and designed the experiments. Y-YD and F-QK contributed for the sampling. TZ, Y-YD, and F-QK performed the experiments and analyzed the data. TZ and Z-XQ wrote the manuscript. All of authors reviewed the manuscript.

## Conflict of Interest

F-QK, Y-YD, and CG were employees of Johnson & Johnson at the time this study was conducted. The remaining authors declare that the research was conducted in the absence of any commercial or financial relationships that could be construed as a potential conflict of interest.

## Footnotes

1 https://www.bioinformatics.babraham.ac.uk/projects/fastqc/

2 http://www.biosino.org/node
