# Supplementary material for: Dynamics of Skin Mycobiome in Infants
Source: Front Microbiol. 2020 Jul 28;11:1790. doi: 10.3389/fmicb.2020.01790 (PMC7401610; doi:10.3389/fmicb.2020.01790)
Supplement: Supplementary file 1 [file Data_Sheet_1.pdf]

Supplementary Material

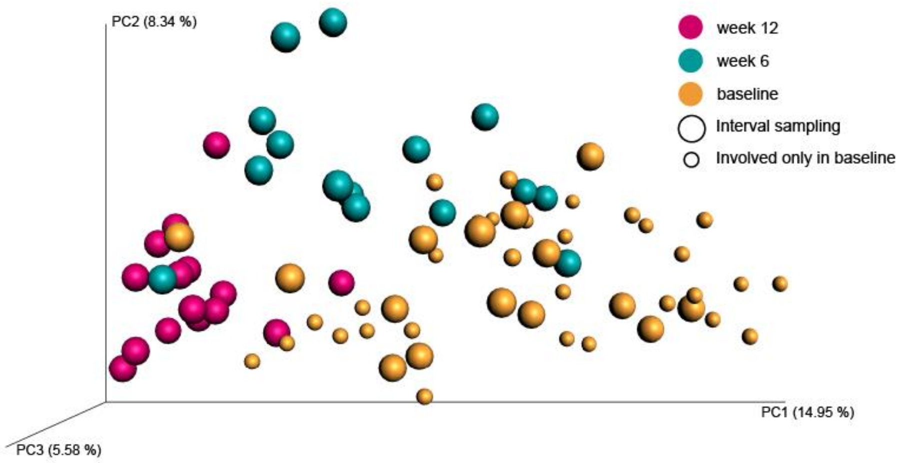

Supplementary Figure S1 | PCoA analysis of the fungal community of 110 infants enrolled in this study. Samples (n = 564) were subsampled according to age, phase, and site. Large circles represent subsamples from 39 subjects included in the three sampling batches (baseline, week 6, and week 12), and small circles represent subsamples from the remainder of the 110 subjects included in only baseline sampling.

Supplementary Table S1 | The information of 110 subjects enrolled in this study. Subjects were all sampled at baseline and 39 of them, with a marker of "3" in the column titled "Sampling times involved in this study", were included in sampling at week 6 and week 12. A1, 0 - 2 months; A2, 2 - 3 months; A3, 3 - 4 months; A4, 4 - 5 months; A5, 5 - 6 months. (Please see Data sheet 2)

Supplementary Table S2 | Proportion of the top 20 fungal genera on each skin site presented in this study

Order	Genus	Back (%, average ± sd)	Antecubital fossa (%, average ± sd)	Volar forearm (%, average ± sd)
**Malasseziales**	***Malassezia***	43.43 ± 28.94	24.48 ± 22.68	41.37 ± 31.99
**Pleosporales**	***Alternaria***	9.85 ± 16.94	14.11 ± 22.52	8.22 ± 17.5
**Capnodiales**	***Cladosporium***	5.43 ± 10.94	9.85 ± 16.97	5.71 ± 14.07
**Saccharomycetales**	***Candida***	6.25 ± 16.14	7.05 ± 15.27	4.42 ± 12.94
**Eurotiales**	***Aspergillus***	1.95 ± 5.88	2.85 ± 7.99	2.58 ± 10.53
**Filobasidiales**	***Cryptococcus***	4.55 ± 14.52	2.28 ± 7.84	2.81 ± 9.99
**Agaricales**	***Schizophyllum***	1.13 ± 4.95	2.58 ± 7.39	0.6 ± 2.83
**Sporidiobolales**	***Rhodotorula***	0.62 ± 2.34	1.95 ± 8.53	1.6 ± 7.52
**Hypocreales**	***Fusarium***	1.82 ± 6.48	1.3 ± 6.51	0.83 ± 3.41
**Eurotiales**	***Penicillium***	0.73 ± 5.45	0.41 ± 1.47	2.09 ± 10.02
**Helotiales**	***Sclerotinia***	0.19 ± 1.15	0.83 ± 7.34	2.09 ± 9.74
**Hypocreales**	***Engyodontium***	1.02 ± 8.81	0.88 ± 7.22	1.16 ± 8.34
**Sordariales**	***Gelasinospora***	1.32 ± 6.75	0.3 ± 1.26	1.03 ± 7.34
**Polyporales**	***Coriolopsis***	0.35 ± 1.98	1.05 ± 6.4	0.47 ± 2.28
**Saccharomycetales**	***Meyerozyma***	0.46 ± 2.00	1.02 ± 4.64	0.18 ± 0.7

**Incertae sedis**	***Pyricularia***	0.54 ±4.04	0.91 ±6.13	0.11 ±0.43
**Capnodiales**	***Toxicocladosporium***	0.39 ±3.47	0.04 ±0.05	0.78 ±5.51
**Erysiphales**	***Erysiphe***	0.41 ±1.79	0.34 ±2.33	0.37 ±2.23
**Polyporales**	***Ceriporia***	0.46 ±4.5	0.4 ±3.74	0.11 ±1.03
**Agaricales**	***Coprinopsis***	0.34 ±2.58	0.61 ±3.14	0.02 ±0.08

Supplementary Table S3 | Mantel test of Bray–Cutis matrix and skin physical parameters.

	**Deep layer hydration**		**TEWL**		**pH**		**Moisture**	
	R	*P*	*R*	*P*	*R*	*P*	*R*	*P*
**A1**	-	NS	-	NS	-	NS	-	NS
**A2**	-	NS	-	NS	-	NS	-	NS
**A3**	-	NS	-	NS	-	NS	-	NS
**A4**	-	NS	-	NS	-	NS	-	NS
**A5**	-	NS	-	NS	0.240	0.005	-	NS
**Urban**	-	NS	-	NS	-	NS	-	NS
**Suburban**	-	NS	-	NS	-	NS	-	NS
**Female**	-	NS	-	NS	-	NS	-	NS
**Male**	-	NS	-	NS	-	NS	-	NS
**Cesarean section**	-	NS	-	NS	-	NS	-	NS
**Vaginal delivery**	-	NS	-	NS	-	NS	-	NS

A1, 0 - 2 months; A2, 2 - 3 months; A3, 3 - 4 months; A4, 4 - 5 months; A5, 5 - 6 months; TEWL, transepidermal water loss; NS, non-significant.

Supplementary Table S4 | ANOVA results for 15 fungal orders presented in Fig. 1A between different sampling times.

Order	*P* value of label pairs			Proportion			Genus in ANOVA analysis
	Baseline *vs.* Week 6	Baseline *vs.* Week 12	Week 6 *vs.* Week 12	Baseline	Week 6	Week 12	
**Malasseziales**	0.00	0.00	0.00	0.36	0.24	0.13	*Malassezia*
**Pleosporales**	NS	NS	0.02	0.16	0.14	0.23	*Alternaria*
**Capnodiales**	NS	NS	NS	0.10	0.13	0.11	*Cladosporium*
**Saccharomycetales**	NS	NS	NS	0.06	0.07	0.10	*Candida*
**Eurotiales**	NS	0.01	0.00	0.05	0.02	0.10	*Aspergillus*
**Hypocreales**	NS	NS	NS	0.03	0.04	0.06	*Fusarium*
**Filobasidiales**	0.00	NS	0.00	0.01	0.10	0.02	*Filobasidium*
**Sporidiobolales**	NS	NS	NS	0.03	0.06	0.04	*Rhodotorula*
**Agaricales**	NS	NS	NS	0.03	0.03	0.02	*Schizophyllum*

**Erysiphales**	NS	NS	NS	0.00	0.02	0.03	*Erysiphe*
**Dothideales**	NS	NS	NS	0.01	0.01	0.02	-
**Trichosphaeriales**	NS	NS	NS	0.02	0.01	0.01	-
**Agaricostilbales**	NS	0.01	0.01	0.00	0.00	0.03	*Sterigmatomyces*
**Sordariales**	0.04	NS	NS	0.02	0.00	0.01	*Gelasinospora*
**Polyporales**	NS	NS	NS	0.01	0.00	0.01	-

NS, non-significant.
