# Supplementary material for: Dynamics of Skin Mycobiome in Infants
Source: Front Microbiol. 2020 Jul 28;11:1790. doi: 10.3389/fmicb.2020.01790 (PMC7401610; doi:10.3389/fmicb.2020.01790)
Supplement: Supplementary file 2 [file Data_Sheet_2.PDF]

Subject ID	Age Group	Age (Days)	Residence	Gender	Delivery type	Sampling times involved in this study	Birth Place	Staying in Beijing all the time?	Pets
B001	A1	46	URBAN	FEMALE	Vaginal Delivery		Beijing	YES	Dog; No contact with baby
B002	A1	45	URBAN	FEMALE	Vaginal Delivery		Beijing	YES	Rabbit; No contact with baby
B005	A1	49	SUBURBAN	MALE	Vaginal Delivery		Beijing	YES	NO
B006	A1	38	URBAN	FEMALE	Vaginal Delivery		Beijing	YES	NO
B007	A1	34	URBAN	MALE	Vaginal Delivery		Beijing	YES	NO
B008	A2	86	SUBURBAN	MALE	Cesarean section		Beijing	YES	Dog and cat; No contact with baby
B011	A2	67	URBAN	FEMALE	Vaginal Delivery		Beijing	YES	NO
B014	A2	77	URBAN	FEMALE	Vaginal Delivery		Beijing	YES	NO
B016	A1	45	SUBURBAN	FEMALE	Vaginal Delivery	3	Beijing	YES	NO
B018	A2	64	SUBURBAN	FEMALE	Cesarean section	3	Beijing	YES	NO
B019	A5	182	URBAN	MALE	Cesarean section		Beijing	YES	NO
B020	A4	122	URBAN	MALE	Cesarean section		Beijing	YES	NO
B022	A5	176	URBAN	FEMALE	Vaginal Delivery		Beijing	YES	NO
B025	A4	134	URBAN	FEMALE	Vaginal Delivery		Beijing	YES	NO
B026	A1	37	URBAN	FEMALE	Cesarean section	3	Beijing	YES	NO
B027	A4	123	URBAN	MALE	Cesarean section		Beijing	YES	NO
B028	A5	177	URBAN	FEMALE	Vaginal Delivery		Beijing	YES	NO
B029	A1	40	URBAN	FEMALE	Cesarean section		Beijing	YES	Dog; No contact with baby
B030	A4	124	URBAN	FEMALE	Vaginal Delivery		Beijing	YES	NO
B031	A5	176	URBAN	MALE	Vaginal Delivery		Beijing	YES	NO
B032	A5	192	URBAN	FEMALE	Cesarean section	3	Beijing	YES	NO
B033	A5	176	SUBURBAN	FEMALE	Cesarean section		Beijing	YES	Dog; No contact with baby
B034	A5	173	SUBURBAN	FEMALE	Vaginal Delivery	3	Beijing	YES	Dog; No contact with baby
B035	A2	72	URBAN	MALE	Vaginal Delivery		Beijing	YES	NO
B036	A4	145	SUBURBAN	MALE	Vaginal Delivery		Beijing	YES	NO
B037	A5	179	URBAN	MALE	Cesarean section		Beijing	YES	NO
B038	A4	135	URBAN	MALE	Vaginal Delivery		Beijing	YES	NO
B041	A5	167	URBAN	MALE	Cesarean section		Beijing	YES	NO
B042	A5	178	URBAN	MALE	Vaginal Delivery		Beijing	YES	NO
B043	A4	125	URBAN	FEMALE	Vaginal Delivery		Beijing	YES	NO
B044	A3	119	SUBURBAN	MALE	Cesarean section		Beijing	YES	NO
B045	A4	135	URBAN	FEMALE	Cesarean section		Beijing	YES	NO
B047	A1	50	URBAN	MALE	Vaginal Delivery		Beijing	YES	NO
B048	A4	137	URBAN	FEMALE	Cesarean section		Beijing	YES	NO
B049	A4	145	URBAN	FEMALE	Cesarean section		Beijing	YES	NO
B051	A1	58	URBAN	FEMALE	Vaginal Delivery		Beijing	YES	NO
B052	A2	88	URBAN	FEMALE	Cesarean section	3	Beijing	YES	Dog; No contact with baby
B053	A5	153	URBAN	FEMALE	Vaginal Delivery		Beijing	YES	NO
B055	A5	181	SUBURBAN	MALE	Cesarean section		Beijing	YES	NO
B056	A4	135	URBAN	MALE	Cesarean section		Beijing	YES	NO
B058	A5	161	URBAN	FEMALE	Vaginal Delivery	3	Beijing	YES	NO
B059	A4	135	URBAN	FEMALE	Vaginal Delivery	3	Beijing	YES	Dog; No contact with baby
B060	A2	85	URBAN	FEMALE	Vaginal Delivery		Beijing	YES	Dog; No contact with baby
B061	A5	163	URBAN	MALE	Cesarean section	3	Beijing	YES	NO
B062	A1	39	URBAN	MALE	Cesarean section	3	Beijing	YES	NO
B063	A5	183	URBAN	MALE	Vaginal Delivery		Beijing	YES	Dog; No contact with baby

B064	A1	44	URBAN	MALE	Vaginal Delivery	3	Beijing	YES	NO
B065	A2	74	URBAN	FEMALE	Cesarean section		Beijing	YES	NO
B067	A3	93	URBAN	FEMALE	Vaginal Delivery	3	Beijing	YES	NO
B068	A3	107	URBAN	MALE	Vaginal Delivery	3	Beijing	YES	NO
B069	A3	102	URBAN	FEMALE	Vaginal Delivery	3	Beijing	YES	NO
B071	A3	94	URBAN	MALE	Cesarean section		Beijing	YES	NO
B072	A4	134	URBAN	MALE	Cesarean section		Beijing	YES	NO
B073	A5	184	SUBURBAN	MALE	Vaginal Delivery	3	Hengshui	Return to Beijing at one month after birth	NO
B075	A5	177	URBAN	FEMALE	Vaginal Delivery	3	Beijing	YES	NO
B076	A4	122	URBAN	MALE	Vaginal Delivery		Beijing	YES	NO
B078	A5	180	SUBURBAN	MALE	Vaginal Delivery	3	Beijing	YES	NO
B079	A5	154	URBAN	FEMALE	Vaginal Delivery	3	Beijing	YES	NO
B080	A1	27	URBAN	FEMALE	Vaginal Delivery		Beijing	YES	NO
B081	A2	61	SUBURBAN	MALE	Cesarean section	3	Beijing	YES	Dog; No contact with baby
B082	A3	113	URBAN	FEMALE	Vaginal Delivery	3	Beijing	YES	NO
B083	A2	64	SUBURBAN	MALE	Cesarean section		Beijing	YES	NO
B084	A1	57	SUBURBAN	MALE	Vaginal Delivery		Beijing	YES	NO
B085	A1	58	SUBURBAN	MALE	Cesarean section		Beijing	YES	NO
B086	A2	67	SUBURBAN	FEMALE	Vaginal Delivery	3	Beijing	YES	Dog; No contact with baby
B087	A5	180	URBAN	FEMALE	Cesarean section	3	Beijing	YES	NO
B088	A1	47	URBAN	MALE	Vaginal Delivery		Beijing	YES	NO
B089	A2	75	URBAN	MALE	Vaginal Delivery	3	Beijing	YES	NO
B090	A5	165	URBAN	FEMALE	Cesarean section	3	Beijing	YES	NO
B091	A1	42	URBAN	MALE	Cesarean section	3	Beijing	YES	NO
B092	A5	160	URBAN	FEMALE	Cesarean section		Beijing	YES	Dog; No contact with baby
B094	A5	165	URBAN	FEMALE	Vaginal Delivery	3	Beijing	YES	NO
B095	A3	106	URBAN	MALE	Cesarean section		Beijing	YES	Dog; No contact with baby
B096	A2	85	URBAN	MALE	Cesarean section		Beijing	YES	NO
B097	A4	139	URBAN	FEMALE	Cesarean section	3	Beijing	YES	NO
B100	A1	46	URBAN	FEMALE	Cesarean section		Beijing	YES	NO
B102	A4	145	URBAN	MALE	Vaginal Delivery		Beijing	YES	NO
B103	A2	71	SUBURBAN	MALE	Cesarean section	3	Beijing	YES	NO
B104	A5	178	URBAN	FEMALE	Cesarean section	3	Beijing	YES	NO
B105	A5	172	URBAN	FEMALE	Cesarean section		Beijing	YES	NO
B107	A2	65	URBAN	FEMALE	Vaginal Delivery	3	Beijing	YES	Dog; No contact with baby
B108	A5	164	URBAN	MALE	Vaginal Delivery		Beijing	YES	NO
B109	A3	95	URBAN	MALE	Vaginal Delivery		Beijing	YES	NO
B110	A4	135	SUBURBAN	MALE	Cesarean section		Beijing	YES	NO
B111	A4	127	SUBURBAN	FEMALE	Cesarean section		Beijing	YES	NO
B112	A2	75	SUBURBAN	MALE	Vaginal Delivery	3	Beijing	YES	NO
B115	A1	49	SUBURBAN	FEMALE	Vaginal Delivery	3	Beijing	YES	NO
B117	A5	174	URBAN	MALE	Vaginal Delivery		Beijing	YES	Dog; No contact with baby
B118	A4	137	URBAN	MALE	Cesarean section		Beijing	YES	NO
B119	A3	114	URBAN	MALE	Cesarean section		Beijing	YES	NO
B122	A3	99	URBAN	FEMALE	Vaginal Delivery		Beijing	YES	NO
B124	A4	139	URBAN	MALE	Vaginal Delivery	3	Beijing	YES	NO

B126	A5	184	URBAN	MALE	Cesarean section	3	Beijing	YES	NO
B127	A4	136	URBAN	FEMALE	Cesarean section		Zhangjiakou	Return to Beijing at two month after birth	NO
B128	A1	60	URBAN	MALE	Cesarean section		Beijing	YES	NO
B129	A2	70	SUBURBAN	MALE	Cesarean section	3	Beijing	YES	NO
B130	A2	68	SUBURBAN	MALE	Cesarean section		Beijing	YES	Dog; No contact with baby
B131	A1	60	SUBURBAN	FEMALE	Vaginal Delivery	3	Beijing	YES	Dog; No contact with baby
B132	A2	65	SUBURBAN	FEMALE	Cesarean section		Beijing	YES	Dog; No contact with baby
B133	A1	59	SUBURBAN	MALE	Cesarean section	3	Beijing	YES	NO
B134	A2	65	SUBURBAN	MALE	Cesarean section		Beijing	YES	NO
B136	A1	57	URBAN	FEMALE	Vaginal Delivery	3	Beijing	YES	NO
B137	A1	48	SUBURBAN	MALE	Vaginal Delivery	3	Beijing	YES	Cat; No contact with baby
B140	A1	38	SUBURBAN	FEMALE	Vaginal Delivery		Beijing	YES	NO
B141	A2	68	SUBURBAN	MALE	Vaginal Delivery		Beijing	YES	NO
B143	A2	67	SUBURBAN	MALE	Cesarean section		Beijing	YES	NO
B146	A2	65	SUBURBAN	MALE	Cesarean section		Beijing	YES	Dog; No contact with baby
B147	A5	154	URBAN	MALE	Cesarean section		Beijing	YES	NO
B148	A5	160	SUBURBAN	FEMALE	Vaginal Delivery		Beijing	YES	NO
B149	A5	152	URBAN	MALE	Cesarean section		Beijing	YES	Dog; No contact with baby
